# The Effectiveness and Cost of an Intervention to Increase the Provision of Preventive Care in Community Mental Health Services: Protocol for a Cluster-Randomized Controlled Trial

**DOI:** 10.3390/ijerph19053119

**Published:** 2022-03-07

**Authors:** Caitlin Fehily, Emma McKeon, Tegan Stettaford, Elizabeth Campbell, Simone Lodge, Julia Dray, Kate Bartlem, Penny Reeves, Christopher Oldmeadow, David Castle, Sharon Lawn, Jenny Bowman

**Affiliations:** 1 School of Psychological Sciences, The University of Newcastle, Callaghan, NSW 2308, Australia; emma.mckeon@uon.edu.au (E.M.); tegan.stettaford@uon.edu.au (T.S.); libby.campbell@health.nsw.gov.au (E.C.); simone.lodge@newcastle.edu.au (S.L.); julia.dray@newcastle.edu.au (J.D.); kate.bartlem@newcastle.edu.au (K.B.); jenny.bowman@newcastle.edu.au (J.B.); 2 Hunter Medical Research Institute, Clinical Research Centre, New Lambton Heights, NSW 2305, Australia; penny.reeves@hmri.org.au (P.R.); christopher.oldmeadow@hmri.org.au (C.O.); 3 Hunter New England Population Health, Wallsend, NSW 2287, Australia; 4 School of Medicine and Public Health, The University of Newcastle, Callaghan, NSW 2308, Australia; 5 Centre for Addictions and Mental Health, Toronto, ON M6J 1H4, Canada; david.castle@camh.ca; 6 Department of Psychiatry, University of Toronto, Toronto, ON M5S 1A1, Canada; 7 Lived Experience Australia Ltd., Adelaide, SA 5001, Australia; sharon.lawn@flinders.edu.au; 8 College of Medicine & Public Health, Flinders University, Belford Park, SA 5042, Australia

**Keywords:** practice change, clinical practice change, mental health services, community mental health, preventive care, smoking, nutrition, alcohol, physical activity, overweight

## Abstract

Preventive care to address chronic disease risk behaviours is infrequently provided by community mental health services. In this cluster-randomised controlled trial, 12 community mental health services in 3 Local Health Districts in New South Wales, Australia, will be randomised to either an intervention group (implementing a new model of providing preventive care) or a control group (usual care). The model of care comprises three components: (1) a dedicated ‘healthy choices’ consultation offered by a ‘healthy choices’ clinician; (2) embedding information regarding risk factors into clients’ care plans; and (3) the continuation of preventive care by mental health clinicians in ongoing consultations. Evidence-based implementation strategies will support the model implementation, which will be tailored by being co-developed with service managers and clinicians. The primary outcomes are client-reported receipt of: (1) an assessment of chronic disease risks (tobacco smoking, inadequate fruit and vegetable consumption, harmful alcohol use and physical inactivity); (2) brief advice regarding relevant risk behaviours; and (3) referral to at least one behaviour change support. Resources to develop and implement the intervention will be captured to enable an assessment of cost effectiveness and affordability. The findings will inform the development of future service delivery initiatives to achieve guideline- and policy-concordant preventive care delivery.

## 1. Introduction

Mental health conditions are a clinically diagnosable disorder that impacts a person’s thinking, feeling, behaviour or mood [1]. The term covers a range of diagnoses (such as mood disorders, anxiety disorders and psychoses and severities) and may lead to people presenting to a mental health service for treatment [1]. A higher prevalence of key chronic disease risk factors (tobacco smoking, inadequate fruit and vegetable consumption, harmful alcohol use, physical inactivity and overweight/obesity [2]) contributes substantially to the greater burden of chronic disease morbidity and mortality [3] experienced by people with a mental health condition. Internationally, the median gap in life expectancy is 10 years, which is evident across the range of mental health conditions [4]. In Australia, the average gap in life expectancy for people receiving treatment from a psychiatric facility, compared to the general population, is 12–16 years, with approximately three quarters due to potentially preventable chronic diseases, such as cardiovascular disease and type 2 diabetes [5]. The need to redress the physical health inequity experienced by people with a mental health condition, and specifically the higher prevalence of chronic disease risk factors, is a recognised priority internationally [6] and in Australia [7].

Clinical practice guidelines [8] and policies [9] direct health services, including mental health services, to provide evidence based ‘preventive care’ to support clients in making positive changes to chronic disease risk behaviours [10,11,12,13]. In Australia, community mental health services, which provide support to the large majority of people who receive specialist mental health treatment, represent a key opportunity to provide preventive care to a large proportion of people with a mental health condition and particularly those with severe mental ill-health [14]. Evidence-based frameworks to guide such care include the ‘AAR’ framework: *assessment* of chronic disease risk factors, *advice* to modify identified risks, and *referral* to support services, providers or groups to provide ongoing behaviour change support [15,16,17]. Preventive care can also include broader elements of assisting clients to change, such as goal setting, motivational interviewing, agency building and follow-up support [18,19], aligning with the frequent and often extended period of engagement with clients that typifies the support provided by multidisciplinary community mental health teams. Research has found that community mental health clinicians do consider that providing preventive care is an important part of their role [20]. Despite this, a meta-analysis of 38 studies indicated that preventive care was infrequently provided by mental health services, including community mental health services [21]. Further, a number of barriers to the provision of preventive care in this setting have been identified, including lack of knowledge regarding risk factors and confidence in assessing them, inadequate time to undertake a comprehensive risk assessment and advice provision within a typical consultation, and a perceived lack of referral options [22].

Research has been conducted to examine the effectiveness of interventions to increase preventive care provision in mental health settings. A recent systematic review by Fehily et al. (2020) identified twenty studies (of any design with a comparison group) with such an aim [23]. Of the included studies (*n* = 20), most examined smoking only (*n* = 14), with few targeting other risk behaviours (*n* = 6). Effective intervention strategies (defined as demonstrating a significant increase in preventive care in at least two studies) were identified within two categories of the Effective Practice and Organisation of Care Taxonomy [24]: (1) delivery arrangements: changes in who is responsible for care provision or how, when or where care is delivered (including embedding additional staff with specialist roles); and (2) implementation strategies: strategies to influence the behaviour of organisations or clinicians, or use of health services by clients (including clinician training, educational materials, health information systems, local consensus processes, authority and accountability, and reminders).

Embedding an additional provider in a mental health service with a dedicated preventive care role is a delivery arrangement with the potential to overcome the aforementioned barriers. The above systematic review identified that this approach led to significant increases in client receipt of preventive care [23], and additional studies have reported this approach increased the provision of broader physical health care (e.g., metabolic outcomes, self-examination, and access to primary medical care) [25]. Both clients [26] and mental health clinicians [27] show support for such an approach, though have indicated the importance of it being coordinated with usual care provision and not adding to the existing fragmentation of physical and mental health care delivery [26]. In our first study exploring this model of care—and the only published study to do so with a focus specifically on multiple key chronic disease risk factors—we undertook a randomised controlled trial with clients (*N* = 811) of one New South Wales (NSW) community mental health service. Clients were randomised to receive either usual care, or usual care plus the offer of an additional consultation with a ‘preventive care clinician’ [28]. At 1-month follow-up, clients in the intervention condition reported significantly greater increases in the receipt of assessment for all four risk factors (*p* < 0.001), advice for all applicable risks (*p* = 0.019), and offers of referrals to applicable telephone support services (*p* = 0.004), as compared to participants receiving usual care. The trial demonstrated that comprehensive AAR care for multiple risks was feasible to offer and deliver in a dedicated consultation within this setting, and further that this could be achieved at a low per-client cost [29]; additionally, it was viewed by clients as acceptable [30]. However, a limitation of the model was noted to be the risk of perpetuating the problematic separation of responsibilities and roles in providing care for physical and mental health (between the preventive care clinician and other mental health clinicians). In this trial, other than a summary recorded by the specialist clinician in the electronic records, the additional consultation was not integrated into clients’ ongoing care. Furthermore, there was no defined role for mental health care managers to continue or reinforce the preventive care provided by the specialist clinician.

Therefore, whilst the findings were largely positive, further research is needed to address this limitation and develop the model of care, specifically with respect to integrating the additional consultation within routine service provision, and identifying implementation strategies that can effectively support services and clinicians. Cochrane systematic review evidence supports the effectiveness of various implementation strategies in increasing preventive care in general health settings, including leadership and consensus processes [31], enabling systems, training [32], audit and feedback [33], and client activation [34]. Only one study has explored the effectiveness of implementation strategies in increasing preventive care provision by clinicians in mental health settings for the key chronic disease risk behaviours of tobacco smoking, inadequate fruit and vegetable consumption, harmful alcohol use and physical inactivity [35]. In a multiple baseline trial (*n* = 19 community mental health services), a suite of the implementation strategies supported by the Cochrane review evidence were trialled to support preventive care provision. The results indicated a significant increase for only one of 16 preventive care outcomes, and it was suggested that the use of generic implementation strategies that were not tailored to the specific context of mental health settings may have contributed to the limited effect [35].

To address this evidence gap, the present study will assess the effectiveness of a model of care in increasing the provision of preventive care for key chronic disease risk factors (tobacco smoking, inadequate fruit and vegetable consumption, harmful alcohol consumption, physical inactivity, and overweight/obesity) in community mental health services. The model consists of three components: (1) offering clients a preventive care consultation (content framed around the AAR framework) with a dedicated ‘healthy choices’ clinician (HCC); (2) embedding information regarding client chronic disease risks into the existing care management plan; and (3) the continued provision of preventive care by mental health clinicians in ongoing consultations. Strategies to support the implementation of the model of care draw on evidence-based implementation strategies [31,32,33,34], tailored to the service settings through co-development with managers and clinicians to consider the clinical, professional and organisational factors of community mental health service delivery. The primary outcomes are client-reported receipt of preventive care (elements within the AAR framework). Client risk factors and attempts to change risk factors, confidence, and readiness to change are assessed as secondary outcomes. Cost and cost-effectiveness of the intervention will also be quantified.

## 2. Materials and Methods

### 2.1. Study Design and Setting

A cluster randomised controlled trial with two parallel groups will be undertaken, with community mental health services as the unit of randomisation. A type 3 hybrid implementation-effectiveness design will be employed, combining both preventive care delivery outcomes and client behaviour change outcomes [36]. Twelve services providing community-based mental health care to adults with a range of mental health conditions will take part, across three local health districts (LHDs) in NSW Australia (encompassing major city, regional and remote areas) [14,37]. Mental health care within these services is generally provided to clients through consultations (of varying number and frequency) with an allocated clinician. Mental health teams are multidisciplinary, and mental health clinicians can be from a variety of disciplines, including psychiatrists, mental health nurses, and allied health staff. In NSW, Australia, state-level policy requires all such services to provide preventive care to address clients’ chronic disease risk factors; however, previous research in parts of this region have found this care is infrequently provided [38,39].

**Figure 1:**
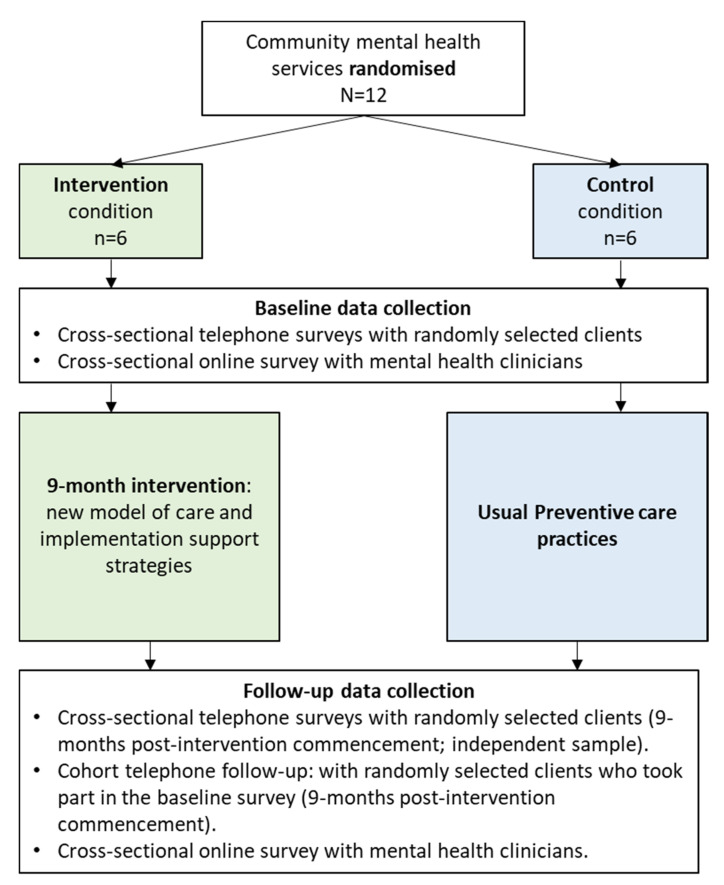
Study design.

Services will be randomised (1:1) to either a 9-month intervention to build their capacity to provide preventive care (comprising the model of care and implementation support strategies) or a usual care control condition. Assessment of preventive care receipt will occur through two independent cross-sectional client telephone surveys, at baseline (primary outcomes) and follow-up (post-intervention). A nested ‘cohort follow-up’ at post-intervention, involving a random sub-sample of clients who participated in the baseline survey, will be used to evaluate client behaviour change outcomes (secondary outcomes; see Figure 1 for study design). Online surveys with mental health clinicians will also be conducted at baseline and follow-up to measure clinician provision of preventive care, implementation process measures (follow-up intervention group only), and resources available to provide preventive care. Intervention and data collection recording tools will capture the adaptations made to the intervention, including any in response to COVID-19. Data will inform an assessment of the intervention cost and cost-effectiveness.

The study was registered with the Australian and New Zealand Clinical Trials Registry: ACTRN12621000922875 (date registered: 15 July 2021). It was approved by the Hunter New England Human Research Ethics Committee (Ref: 2020/ETH03234) and the University of Newcastle Human Research Ethics Committee (Ref: H-2021-015), and authorised for conduct by the Central Coast (Ref: 2021/STE00265) and Mid North Coast (Ref: 2021/STE00264). Supplementary-Table S1 contains the Standard Protocol Items: Recommendations for Intervention Trials (SPIRIT) checklist, noting where all recommended items were reported in this protocol.

### 2.2. Randomisation and Blinding

Twelve services will take part in the study. Based on the number of services available in each LHD, this will include six from one LHD, three from the second, and three from the third. An independent statistician will use a computerised random number function to randomly allocate services in a 1:1 ratio to either the intervention or control condition within the strata (three LHDs). Constrained randomisation will be used to ensure balance between the intervention and control groups in terms of service characteristics: size (based on client numbers; 2 levels; cut off point of 1500), structure (2 levels: single site versus multisite), and rurality (determined by service postcode; 2 levels: major city vs. inner/outer regional [40]). Following the randomisation, researchers, services, clinicians, and clients will not be blind to allocation. Statisticians undertaking outcome analyses will be blinded to group allocation. Interviewers collecting data from clients will be blinded to group allocation (with the exception of the end of the follow-up survey where intervention participants will be asked additional questions regarding the intervention).

### 2.3. Participant Eligibility and Recruitment

#### 2.3.1. Community Mental Health Services

Agreement for an LHD to participate in the trial was sought from the mental health director for each LHD. Community mental health services within each LHD were eligible to take part if they were not currently engaged in other research with the team. The identification of discrete services (‘clusters’) to be included in the trial was then determined through an assessment of management structure (e.g., services having their own manager), size (annual client throughput and number of clinical staff) and structure (single site or multisite). Within two LHDs, smaller sites that came under one overarching manager were treated as one service for this trial (considered multisite services). In the other LHD, it was not possible to ensure that different services had unique managers, as two managers oversee staff across multiple locations, including both intervention and control services. However, the services have discrete clinicians and clients.

#### 2.3.2. Clients

Participants will be clients of the participating services. Participation in data collection will be independent of any care provided by the service. Data collection via computer-assisted telephone interviews (CATI) will be conducted through cross-sectional surveys (Figure 1) with eligible clients (outlined below).

##### Cross-Sectional Surveys

Clients of intervention and control services who are 18 years or older and attended at least two consultations in the previous 9 months will be eligible to be randomly selected (from service records) for participation in the two independent cross-sectional surveys (baseline and follow-up). An average of 120 eligible clients per service will be randomly selected from the electronic health service records during both the baseline and follow-up period (720 intervention group; 720 control group). These clients will be mailed an information statement by the service describing the nature of the study and providing an opportunity to opt-out by calling a toll-free number. Clients who do not opt-out will be contacted 2 weeks later by trained telephone interviewers within a NSW health service CATI team to assess the additional eligibility criteria (English speaking (due to practical constraints); mentally and physically capable of responding to survey items; and does not report having an eating disorder as their primary mental health condition) and seek their consent to participate. Clients could be randomly selected as part of both baseline and follow-up samples, with consent to take part in each survey sought independently.

##### Cohort Client Follow-Up (Nested Study)

A random selection of eligible client participants (100 from each group) will form a cohort sub-sample who will be contacted via telephone and invited to complete the cohort follow-up survey. Eligible clients will be those who completed the baseline survey and, at that time, consented to be contacted regarding the cohort follow-up; and either (1) were offered the healthy choices consultation (if in the intervention group) or (2) had at least one mental health consultation in the preceding 9 months (if in the control group).

#### 2.3.3. Mental Health Clinicians

It is intended that all clinicians (psychiatrists, psychologists, social workers, peer workers, nurses, and other disciplines) working in the services allocated to the intervention group received the intervention. Clinicians of intervention and control services who provide direct clinical care to clients and are over 18 will be eligible to take part in online surveys on two occasions: baseline and follow-up (cross-sectional surveys, independent samples). At each data collection point, clinicians will be sent an email from their service manager providing the information statement and containing a link to the survey if wish to complete it (approximately 20 clinicians per service on average). The initial survey questions will confirm clinician eligibility.

### 2.4. Intervention

**Figure 2:**
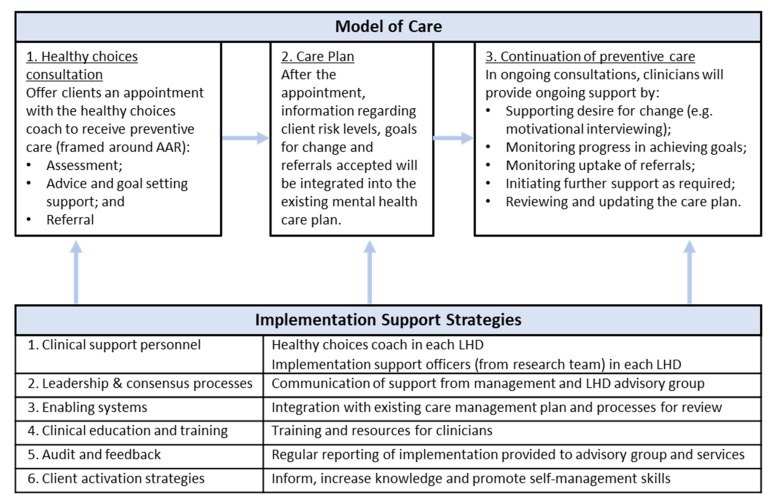
Outline of the intervention: model of care and implementation strategies.

A multi-component clinical practice change intervention will be implemented in the intervention group services for 9 months. This timeframe was chosen based on consultation with participating LHDs for logistical reasons (i.e., enabling delivery of the multi-component intervention). The intervention will involve two parts: (a) a model of providing preventive care; and (b) implementation strategies to support model implementation. Figure 2 displays these two parts, and they are described in further detail in Section 2.4.2.

The key elements of the model of care and implementation strategies were pre-determined by the research team based on relevant evidence [15,16,17,31,32,33,34]. These key elements are also displayed in Figure 2. These elements will provide a foundation for the co-development process (described below), which will explore how these may be implemented.

#### 2.4.1. Co-Development Process

At project commencement, a working group for each LHD will be established, with representation from the research team, service managers and clinicians. These working groups will aid in tailoring the intervention delivery to each service’s usual procedures and to local availability of behaviour change support options. A series of initial meetings will be conducted to provide input into intervention design. This will include reviewing the described model of care pre-determined by the research team (to ensure integration with usual service delivery), with a particular focus on the implementation support, to consider how the implementation strategies will be designed and delivered.

Table 1 provides an overview of the pre-determined elements of the intervention that will provide a foundation for co-development and examples of elements that will be determined through co-development. The tailoring of the model of care and implementation strategies will occur predominantly at the LHD level (e.g., how/where information about client risk factors will be embedded into the existing care management plan); however, some tailoring may be necessary at the service level due to unique service characteristics (e.g., delivery of clinician training within existing training opportunities that are unique to each service). The model of care and implementation support are flexible in terms of delivery to allow for changes that could be needed due to COVID-19 restrictions on service delivery or general restrictions. The working groups will continue to provide advice and guidance throughout the 9-month intervention period.

During the development and implementation phases, input will be sought from Aboriginal representatives to ensure intervention development and implementation is culturally appropriate, as well as mental health consumer and peer-worker representatives to appropriately consider consumer experiences and perspectives. This will firstly be undertaken through separate meetings with these representatives (identified by the participating LHDs) or with the existing consultative groups that exist in some of the participating LHDs. Following this, the research team will ask representatives their preferred way to continue to contribute to intervention development and implementation. This could include membership on the working group, through seeking advice separately, e.g., during existing meetings of consultative groups within the LHDs. Feedback will also be sought on the client activation materials (described in Table 1).

#### 2.4.2. Model of Preventive Care

The model of care consists of three key components, which will be delivered to clients of mental health services during the 9-month intervention period (see Figure 2 for summary):

- ‘Healthy choices’ consultation

A 40 minute consultation dedicated to addressing chronic disease risk factors and provided by a ‘Healthy Choices Clinician’ (HCC) will be offered to adult clients. The consultation will be framed around the evidence-based AAR framework [15,16,17] for key chronic disease risk factors (smoking, nutrition, alcohol, physical activity, and overweight/obesity) and collaboratively setting behaviour change goals with the client. The consultation will incorporate evidence-based behaviour change techniques, such as motivational interviewing [41]. The HCC could have a range of professional backgrounds (e.g., occupational therapist, dietician, nurse, and social worker) and will have expertise in health behaviour change strategies. The fractional HCC appointment (hours per week) required in each service will be determined through co-development (proportional to number of clients and staff). HCCs will complete training in intervention delivery according to a manualised protocol. The consultation, tailored to each client, will include:

- Assessment: Clients will be asked about their current engagement in risk factors, with risk status determined in accordance with the Australian National Health and Medical Research Council Guidelines (Table 2) [42,43,44,45,46].
- Brief advice and goal setting: Information will be provided regarding how the client’s risk factors compare to Australian guidelines. Tailored motivational interviewing strategies will be used to foster client desire to change their risks and build agency. Clients will be encouraged to identify at least one health improvement goal in line with their risk status and/or desired areas for improvement.
- Referral: Clients will be offered referrals to services that provide behaviour change support, based on their risk status [42,43,44,45,46] and/or their identified health improvement goals. This could include referral to services, such as state-level telephone services (e.g., the NSW Quitline [47] and NSW Get Healthy Information and Coaching service [48]) and local providers (e.g., GP, dietician, support groups, and walking groups).

ii. Chronic disease risk information embedded in the care plan

Mental health care management plans are an existing part of mental health service delivery for all participating LHDs. Additional content will be embedded into this existing care plan in the client’s electronic record, including the client’s risk factors, goal(s) for change, and any referrals to behaviour change supports (offered and accepted). The plan will serve as a basis for the client’s clinician to provide continued preventive care (described below).

iii. Continuation of preventive care by mental health clinicians

Clinicians will provide ongoing follow-up and support for chronic disease risk factors in their routine consultations. After a client has had a ‘healthy choices’ consultation, the HCC will liaise with the client’s treating mental health clinician regarding the appointment content and outcomes, and the clinician will review the relevant content of the care plan. Commencing at the following scheduled appointment with the client, the clinician will provide ongoing follow-up and support. This will include behaviour change support, such as encouraging and motivating desire for behaviour change, building agency for change and monitoring progress towards goals. Clinicians will encourage connection with referral services, by monitoring uptake of referrals provided and initiating further support as required. Clinicians will regularly review and update the care plan as required. The service will define how clinician review of their clients’ risk behaviour goals and progress will occur on a regular basis.

#### 2.4.3. Implementation Support Strategies

Supported by findings of Cochrane reviews, evidence-based implementation strategies [31,32,33,34] will be provided over a 9-month period to support the intervention services in implementing the model of care as part of routine practice.

Based on the Cochrane review evidence, ongoing stakeholder consultation, and the team’s previous trials [28,49], the following strategies will be implemented (described in detail in Table 1):

- Clinical support personnel: The HCC embedded within each service to support care delivery and provide education to staff. Implementation support officers (members of the research team; one allocated to each LHD) will support HCCs in this role.
- Leadership and consensus processes: Leaders in each LHD (e.g., service managers) will communicate their strong support for the project. Within each LHD, an advisory group will be established to oversee project direction and implementation.
- Enabling systems: Content from the healthy choices consultation will be integrated into the existing mental health care plan, which will be reviewed regularly in clinical review meetings.
- Clinician education and training: Training sessions and resources will be provided to cases managers regarding strategies for supporting client behaviour change, the importance of addressing chronic disease risks and the logistics of model implementation. Training of the HCC in their role.
- Audit and feedback: An update of progress will be prepared by the HCC and discussed in advisory and working groups to review progress and consider recommended actions.
- Client activation strategies: Resources to build behaviour change agency and self-management skills, and to inform clients about the new model of care (e.g., posters in waiting rooms, client diaries, and educational brochures).

### 2.5. Control

Services randomised to the control condition will continue to provide preventive care for chronic disease risk factors in accordance with their usual practices.

### 2.6. Measures

Study measures are described in detail below. In summary, the primary outcomes are client-reported receipt of preventive care (AAR framework) for multiple chronic disease risks. The secondary outcomes are client receipt of individual AAR elements for each chronic disease risk behaviour and weight; conversations with mental health clinician about behaviour change goals; and client health behaviour change measures (risk factor variables and attempts, confidence and readiness to change behaviours). Supplementary-Table S2 contains the SPIRIT schedule: the recommended schematic diagram of the study measures and time periods.

#### 2.6.1. Primary Trial Outcomes

The primary outcomes are the proportion of clients who reported receiving elements of the AAR framework of preventive care: (1) assessment for all chronic disease risk behaviours (tobacco smoking, inadequate fruit and vegetable consumption, harmful alcohol use and physical inactivity), (2) brief advice for all relevant risk behaviours (Table 1), and (3) referral to at least one behaviour change service for a relevant risk behaviour.

#### 2.6.2. Secondary Trial Outcomes

##### Client Receipt of Preventive Care

The secondary outcomes regarding preventive care are client-reported receipt of the individual elements of preventive care for the individual AAR elements for each chronic disease risk behaviour and weight, and conversations with their mental health clinician about their behaviour change goals. Clients will be asked to report their receipt of each preventive care element (assessment, advice and referral) for each individual risk factor (tobacco smoking, inadequate fruit and vegetable consumption, harmful alcohol consumption, physical inactivity, and overweight/obesity) from their mental health service in the previous 9 months (total of 15 items). Participants will also be asked whether their mental health clinician spoke to them in the preceding 9 months about their goals for behaviour change (yes/no/do not know).

##### Client Behaviour Change

Clients will self-report their levels of chronic disease risk factors over the previous month, specifically:

- Whether they smoked any tobacco products (yes, daily; yes, at least once a week; yes, less than once a week; not at all, quit less than 6 months ago; not at all, quit 6 months or more ago; not at all, never smoked; do not know) and how many cigarettes they smoked per day;
- How many serves of fruit (open ended) and vegetables (open ended) they typically consumed per day;
- How often they consumed alcohol in the last month (never, I do not drink, e.g., never have; none in the last month, e.g., nil last month, drinks occasionally; once a month; 2-to-4 times a month; 2-to-3 times a week; 4 or more times a week; do not know). Those who report consuming alcohol in the previous month will be asked to report how many standard drinks they consumed on a typical drinking day (open ended) and how often they consumed five or more standard drinks on one occasion (never; less than monthly; monthly; weekly; almost daily; daily; do not know) (items adapted from the Alcohol Use Disorders Identification Test (AUDIT-C) for Alcohol Use [50]);
- How many minutes they engaged in walking, moderate physical activity, vigorous physical activity, and strength activities in a typical week (items adapted from the International Physical Activity Questionnaire [51]);
- Their current weight (kg) and height (cm).

Participants will also be asked whether they made any attempts to change each of the chronic disease risk factors in the previous 9 months (yes/no/do not know). For each risk factor, participants will be asked to report their readiness to change by selecting which option best describes them: I never think of changing (risk factor); I sometimes think about changing (risk factor); I have decided to change (risk factor); I am already trying to change (risk factor); my (risk factor) has changed [52]. Participants will report on a 1 to 10 scale how confident they are in their ability to make changes relevant to each risk factor [53,54]. For these measures, smoking items will be asked of participants who reported currently smoking and alcohol items of participants who report consuming any alcohol.

#### 2.6.3. Client Sociodemographic Characteristics

Client age, gender, postcode, and number of community mental health appointments within the previous 9 months will be obtained from the service electronic records. In the surveys, participants will report their primary psychiatric diagnosis, highest educational level, employment status and relationship status.

#### 2.6.4. Mental Health Clinician Provision of Preventive Care

In the baseline and follow-up online surveys, staff will report, for each of the risk behaviours, the proportion of their clients who had risk behaviours documented in their care plan and with whom the clinician had a conversation about referral services. Staff will also indicate (on a 5-point Likert scale) how regularly they: check in with clients about their progress in achieving behaviour change goals, check in with clients about their uptake of referral services, and update the care plan as clients progress. Clinicians will report if their opportunity to provide preventive care was impacted by COVID-19.

#### 2.6.5. Implementation Process Outcomes

In the follow-up online survey, staff of intervention services will report the perceived intervention acceptability, appropriateness, and feasibility using validated measures [55]. The delivery and uptake of the implementation strategies will be measured in project logs recorded by the research team/implementation support officers and HCCs, including advisory group meetings held, employment of HCC, implementation support provision (number and mode of interactions), participation of staff in training, provision of audit and feedback summaries, and provision of client resources. Adaptations made to the intervention, including in response to COVID-19 (e.g., constraints on face-to-face service delivery) will be assessed and recorded by the project team in monthly meetings. To capture aspects of the model of care, delivery of the ‘healthy choices’ consultation will be recorded by the HCC using an electronic template (e.g., number of appointments held, provision of care elements, and client risks and goals set). Content within clinical review meetings relating to preventive care will be assessed (likely by the HCC).

#### 2.6.6. Cost Data

The resources required to develop and implement the model of care will be prospectively collected during the intervention period in project logs, such as training for staff, modification of service electronic tools, and resources required to deliver the healthy choices consultation (e.g., HCC salary). In the online staff survey (baseline and follow-up), staff will report professional discipline, role and hours worked per week to valuation of their time. Staff will also report how frequently they provided preventive care and the average time taken to provide preventive care to an individual client.

### 2.7. Sample Size and Power

An average of 120 eligible clients per service will be randomly selected from service records during both the pre- and post-implementation periods (720 intervention group; 720 control group). An estimated 25% of selected clients will consent and complete the interviews, with a total of 360 clients at each time point (180 intervention group; 180 control group). Assuming an intraclass correlation of 0.05 (to adjust for clustering) and a baseline prevalence of 20% [28], this sample size will provide 80% power to detect at least a 25% absolute increase in the primary practice change outcomes, with a type 1 error rate of 0.017 to account for multiple testing.

For the cohort follow-up, it was estimated that 60% [55] of the 200 randomly selected participants will complete the cohort follow-up survey, providing a sample of 120 (*n* = 60 in each group).

### 2.8. Analysis

Mixed effects logistic regression models will compare the primary outcomes and secondary practice change outcomes (preventive care receipt) between the intervention and control groups at the post-implementation follow-up, adjusting for clustering by service with a service level random intercept. The models will also adjust for potential confounders, including baseline prevalence of preventive care, age, gender, psychiatric diagnosis, and length of time in treatment, as well as LHD as fixed effects. Analyses will be carried out on an intention to treat basis.

In the cohort sub-study, generalised mixed effects regression models will determine the change over time (baseline to 9-month cohort follow-up) in the intervention compared to control participants in the measures relating to client behaviour change: smoking status (yes/no), serves of vegetables per day, serves of fruit per day, number of standard alcohol drinks per week, minutes of moderate and vigorous physical activity per week and body mass index (BMI). Models will adjust for clustering by service and potential confounders as well as LHD. Descriptive analyses will be used to report the remaining measures.

### 2.9. Economic Analysis

To provide a measure of resources required to develop and implement the new model of care, the cost of the intervention will be prospectively measured and valued from a health service delivery perspective. Cost-effectiveness analysis will compare the relative costs and effect of the intervention to usual care delivery in terms of trial primary outcomes. Sensitivity analyses may be conducted to determine impact of clinician salaries and COVID-19 (e.g., changes in the number of hours worked per week). Budget impact analysis will estimate the affordability of the intervention, calculating real-world implementation costs in financial terms, considering the specific contexts of each health district.

## 3. Discussion

Recommended preventive care to address chronic disease risk behaviours is infrequently provided by community mental health services. This protocol described the rationale and design of a trial that aimed to determine the effectiveness of a clinical practice change intervention to increase the guideline-concordant delivery of preventive care in community mental health services. The study is one of few aiming to do so with a focus on multiple key chronic disease risk behaviours [23], and the first to test the proposed novel model of care. The results of the trial will provide important information regarding the effectiveness and cost of an intervention to build the capacity of community mental health services to implement evidence-based preventive care for their clients. 

The model of care involves offering clients an additional consultation with a ‘healthy choices’ clinician, embedding information regarding chronic disease risk behaviours into existing care management plans, and continued provision of preventive care by mental health clinicians. This model was developed based on evidence for the effectiveness [28] and cost efficiency [29] of embedding a dedicated preventive care provider in community mental health services, and the acknowledgement that such a dedicated role needs to be integrated with usual mental health care delivery [27]. The implementation of this model of care will be supported by implementation strategies, selected based on Cochrane systematic review evidence for their effectiveness in supporting implementation of health care delivery [31,32,33,34]. Previous research by our team suggests that tailoring these strategies to the specific context of mental health settings may increase their effectiveness [35].

The findings add to the limited literature regarding interventions to support the delivery of preventive care for multiple chronic disease risk behaviours. This knowledge will inform the development of future service delivery initiatives to achieve guideline- and policy-concordant preventive care delivery.

This study has some limitations. Firstly, the recruitment method for the client surveys relies on mail and telephone. This may preclude some people from participating, for example, people who do not have access to a telephone. Secondly, the primary outcomes (client self-reported receipt of preventive care) are subject to sampling and recall bias. The inclusion of additional process outcomes (e.g., staff-reported provision of preventive care; HCC records) will enhance reporting. Providing such preventive care has the potential to improve both physical and mental health outcomes for this population group, with evidence that positive changes to risk behaviours can lead to improved mental health and mental wellbeing [56,57,58,59].

## 4. Conclusions

The proposed study will increase knowledge regarding effective initiatives to support routine preventive care delivery within community mental health services. Identifying effective population-level approaches to reducing the high prevalence of chronic disease risk factors for people with a mental health condition has the potential to prevent chronic disease development and to improve the life expectancy and quality of life of this vulnerable population group.

## Figures and Tables

**Table 1:** Overview of the co-development process.

Component/ Strategy	Description (Providing a Basis for Co-Development)	Examples of Elements to Be Determined Through Co-Development
Model of Care		
Healthy choices consultation	Clients are offered an appointment with the healthy choices clinician (HCC) to receive preventive care (framed around the AAR framework): Assessment. Advice and goal setting support. Referral.	Ways to promote and offer/schedule the consultation. Referral options. Client eligibility criteria (keeping with a universal approach where possible) and prioritising client groups to receive the consultation.
Care plan	After the consultation, information regarding client risks, goals for change, and referrals offered and/or accepted are integrated into the existing mental health care plan.	Where in the care plan such information should be integrated (e.g., under a new/existing heading). Who enters the information into the care plan (HCC or mental health clinician). Additional content to include in the care plan to inform ongoing care.
Continuation of preventive care	In ongoing mental health consultations (commencing in the next scheduled appointment after the healthy choices consultation), clinicians provide ongoing support and follow-up by: Supporting and encouraging change (e.g., motivational interviewing; building behaviour change agency). Monitoring progress in achieving goals. Monitoring uptake of referrals; offering new/additional referrals. Reviewing and updating the care plan.	Key focal points of ongoing preventive care (e.g., following up on referrals, providing further motivational interviewing, adjusting goals). Frequency and duration of this ongoing care. How, when and the frequency with which ongoing preventive care is reviewed (e.g., in clinical review meetings).
Implementation Support Strategies		
Clinical support personnel	The HCC embedded within each service is funded for 9 months by the trial, employed by the LHD as a member of the service. In addition to client care delivery, the HCC also provides support and training to clinicians. Implementation support officers (research team members; one allocated to each LHD) will regularly contact the HCC to support them in this role.	Preferred clinical background and experience of the HCC and the title for the position. The fractional HCC appointment (hours per week) required in each service, proportional to number of clients and staff. Additional duties (e.g., completion of existing tools and involvement in training). Frequency and mode of Implementation Support officer contact with HCC.
Leadership and consensus processes	Managers communicate support for the new model to all clinicians and reinforce its alignment with state policy and the LHD’s strategic directions. An advisory group for each LHD is established, with representation of the research team, directors, and service managers to oversee implementation, and to monitor and provide feedback throughout.	Avenues for leaders to communicate support for the new model of care to clinicians (e.g., email distribution lists, videos, flyers, and staff meetings). Training or resources to provide managers to support them in facilitating implementation. Advisory group members and frequency
Enabling systems	The HCC records a summary of each ‘healthy choices’ consultation in the electronic medical record system. Content regarding risk behaviours, goals for change, and referrals offered and/or accepted is integrated into the existing care management plan that is part of routine mental health care delivery. The review of the plan is incorporated within the regular team clinical review meetings where the needs and progress of individual clients are discussed. The HCC attends clinical review meetings, contributing to discussions of how plans are incorporated in routine care and offering additional support or advice to clinicians as required.	Detail of where/how health choices consultation recorded in electronic medical record. Other changes to electronic systems that may support implementation, e.g., a process to streamline referrals, such as a referral template or compilation of available referral services, and a template for the HCC to record detail of the ‘healthy choices’ consultation content. Formal and informal communication channels between the HCC and clinicians regarding care plan content (e.g., the HCC notifying clinicians when new content is added to care plans). Regular agenda items in clinical review meetings for the HCC to discuss.
Clinician education and training	Training sessions and resources are provided regarding the importance of addressing risk factors for both physical and mental health, risk guidelines and referral services. Clinicians are upskilled in strategies to support client behaviour change, including motivational interviewing, setting and reviewing, e.g., goals, and identifying and addressing barriers. Training is provided in the processes to implement the model of care, such as in scheduling and facilitating client attendance of the ‘healthy choices’ consultation and checking and updating the care plan.	Frequency, length and mode (online and face-to-face) of training for HCCs and clinicians. It is expected that the HCC has a role in training delivery for clinicians. Type of training (interactive, roleplays, self-paced, and recorded webinars). Useful resources for training (manuals, evidence guides, ‘how to’ guides). When training is held (e.g., during existing training opportunities or scheduled as a separate training session).
Audit and feedback	A summary of preventive care delivery and implementation of the intervention is regularly prepared by the HCC and provided to the LHD advisory group to review progress and consider possible implementation modifications. Summary is also provided to managers and clinicians.	Avenues/details for providing feedback (established LHD meetings, email, etc.) and details of who sends this. Content and frequency of reporting. Potential setting of targets to be reported on (e.g., number of healthy choices consultations held).
Client activation strategies	Resources are provided to clients to build behaviour change agency and promote self-management skills, such as educational brochures. Materials are also developed to inform clients about the model of preventive care. New clients receive information outlining the care they will receive as part of standard intake processes.	Types of materials to promote the healthy choices consultation (posters in waiting rooms, brochures, etc.). Types of resources to support self-management, e.g., client diaries and educational brochures. How resources are provided to clients, e.g., HCC and/or clinicians, including resources in intake packs provided to new clients.

**Table 2:** Definition of risk in accordance with the Australian National Guidelines.

Risk Factor	Definition of Risk	References
Tobacco smoking	Any tobacco smoking	[43]
Inadequate fruit and vegetable intake	Consuming less than two servings of fruit or five servings of vegetables daily (as an indicator of poor nutrition)	[44]
Harmful alcohol consumption	Consuming more than two standard drinks on an average day or five or more on any one occasion	[45]
Physical inactivity	Engaging in less than 150 min of moderate-intensity physical activity or 75 min of vigorous-intensity physical activity, or an equivalent combination of each, weekly	[44]
Unhealthy weight	Waist circumference above 80 cm for women and 94 cm for men, or body mass index (method to be determined through co-development)	[46]

## Data Availability

To preserve participant confidentiality and consistent ethics approvals, data are not be publicly available. Data may be made available upon reasonable request to the corresponding author.

## Supplementary Materials

The following supporting information can be downloaded at: https://www.mdpi.com/article/10.3390/ijerph19053119/s1. Table S1: SPIRIT checklist; Table S2: SPIRIT schedule.
